# Effects of Hybrid-Type Multicomponent Exercise Training on Cardiopulmonary Capacity, Resting Metabolic Rate, and Muscle Strength Following Bariatric Surgery

**DOI:** 10.1007/s11695-026-08647-9

**Published:** 2026-05-14

**Authors:** Burke Koksalan, Aslı Nur Bahar, Omer Gunal, Ozgur Kasimay, Nurper Ozbar, Asim Cingi, Dilek Gogas Yavuz, Alexios Batrakoulis, Meral Kucuk Yetgin

**Affiliations:** 1 Department of Physical Education and Sport, Institute of Health Sciences, Marmara University, Istanbul, Türkiye; 2 Department of Sport Management, Faculty of Sport Sciences, Istanbul Aydin University, Istanbul, Türkiye; 3 Department of Sports Physiology, Faculty of Medicine, Marmara University, Istanbul, Türkiye; 4 Department of General Surgery, Department of Surgical Medical Sciences, Faculty of Medicine, Marmara University, Istanbul, Türkiye; 5 Department of Coaching Education, Division of Movement and Training Sciences, Kırkpınar Faculty of Sport Sciences, Trakya University, Edirne, Türkiye; 6 Department of Endocrinology and Metabolism, School of Medicine, Marmara University, Istanbul, Türkiye; 7 Department of Life Sciences,, European University Cyprus, Nicosia, Cyprus; 8 Department of Physical Education and Sport Science,, Democritus University of Thrace, Komotini, Greece; 9 Department of Coaching Education, Sports Health Sciences, Faculty of Sports Sciences, Marmara University, Istanbul, Türkiye; 10 Department of Applied Health Science, School of Public Health, Indiana University, Bloomington, IN USA

**Keywords:** Sleeve gastrectomy, Hybrid exercise, VO_2_peak, Resting metabolic rate, Muscle strength

## Abstract

**Background:**

The aim is to investigate the effects of a hybrid-type multicomponent exercise program on muscle strength, resting metabolic rate (RMR), cardiopulmonary capacity (VO_2_peak), and body composition after bariatric surgery.

**Methods:**

Twenty adults (5 males, 15 females; BMI ≥ 30 kg/m^2^) who underwent sleeve gastrectomy were evenly assigned to a hybrid-type multicomponent exercise group (HEG) or a Control Group (CG). Three months after the surgery, the HEG commenced a hybrid exercise program, which was undertaken three days per week for a period of four months. A range of health and fitness metrics were measured at the beginning of the study and after the intervention, including musclular strength, body composition, skeletal muscle index (SMI), VO2max, and RMR. Exercise effects were analysed using two-way repeated-measures ANOVA, with sex included as a covariate to account for any differences between the groups.

**Results:**

Significant time effects were observed for all anthropometric and body composition variables in both groups (*p* < 0.001), reflecting the dominant effect of surgery. Reductions in body weight and BMI and regional adiposity were greater in the HEG (*p* < 0.01), whereas lean body mass, SMI and RMR decreased similarly in both groups (*p* > 0.05). In contrast, significant time × group interactions with large effect sizes were found for all upper- and lower-extremity strength measures (*p* < 0.001), favoring the HEG, with the greatest gains in the lower extremities (*p* < 0.001). Handgrip strength and relative VO_₂_peak increased significantly only in the HEG (*p* < 0.001). Physical activity levels increased markedly in the HEG (*p* = 0.003), with all participants classified as highly active post-intervention, while the CG largely remained in low or moderate categories (*p* < 0.001).

**Conclusion:**

Although the hybrid-type multicomponent exercise program does not prevent early postoperative reductions in RMR and lean body mass after bariatric surgery, it provides associated with functional and metabolic benefits by improving muscle strength, cardiorespiratory fitness, physical activity levels, and adiposity-related outcomes, thereby supporting functional capacity.

## Key points

The hybrid-type multicomponent exercise program implemented after bariatric surgery significantly enhanced cardiopulmonary capacity and muscle strength, thereby supporting functional recovery.

Resting metabolic rate and fat-free mass did not increase in either group after surgery, and the hybrid exercise intervention was unable to prevent these physiological declines.

Improvements in body weight, body fat percentage, and other anthropometric parameters were observed in both groups, with more pronounced gains in the hybrid exercise group.

## Introduction

Bariatric surgery in people living with obesity has positive effects on body composition, physical function, metabolic parameters, autonomic nervous system modulation, and, to some extent, energy expenditure, physical activity level, muscle strength, and maximal oxygen uptake [1]. Models in which bariatric surgery is supported by lifestyle modifications positively influence the success of the treatment. Regular physical activity is essential for sustaining healthy lifestyle behaviors after bariatric surgery (BS), yet most patients exhibit low activity levels within the first three postoperative years [2]. Inactive individuals experience significant muscle loss (~7.6kg) and a 29.7% reduction in total body weight, associated with decreased muscle strength [3].

In contrast, patients engaging in regular exercise demonstrate improvements in cardiovascular endurance, muscle mass, and strength, alongside reductions in fat mass, which are further enhanced with nutritional therapy [4]. The American College of Sports Medicine recommends at least 250 min of exercise per week or 2000 kcal of energy expenditure to maintain long-term weight loss following BS [5–7]. Most post-BS interventions focus on aerobic training, with limited studies addressing resistance or combined programs [8, 9]. High-intensity interval training improves cardiovascular function and strength [10], whereas resistance exercise prevents muscle loss and promotes fat reduction, emphasizing the value of combining resistance training and protein supplementation in postoperative programs [11]. It is important to note that physical exercise for the purpose of weight reduction, in addition to the aforementioned training modalities, has been recently identified as one of the most prevalent health and fitness trends on both a global and national scale [12, 13].

Hybrid exercise programs, integrating resistance and cardiovascular training within a single session, simultaneously engage musculoskeletal and cardiovascular systems, promote high muscle activation, and enhance adherence and motivation [14–19].

Maintaining muscle mass and resting metabolic rate is critical for effective weight management, as diet-only interventions may lead to muscle loss and metabolic decline [20–22] which physical activity counteracts [23]. According to a recent meta-analysis, exercise performed after bariatric surgery has been found to be effective particularly in reducing body weight, waist circumference, and BMI, but it does not yield significant improvements in body composition. The best outcomes were observed in combined exercise programs and in interventions initiated more than six months after surgery [24].

A hybrid, multicomponent exercise program provides a time-efficient model that activates multiple muscle groups simultaneously, thereby enhancing adherence and motivation. However, there is a limited number of studies in the literature investigating the effects of early, home-based hybrid exercise programs implemented during the rapid weight-loss phase on muscle strength, resting metabolic rate, and body composition. In addition, limited access to exercise facilities often restricts physical activity in individuals living with obesity, highlighting the value and practicality of home-based online hybrid training for patients after bariatric surgery. The intervention was initiated at the third postoperative month, as this period is considered appropriate for introducing structured and progressive exercise following sufficient recovery, while coinciding with the rapid weight-loss phase during which the risk of lean mass loss is elevated. Therefore, the aim of this study was to investigate the effects of a 4-month hybrid, home-based exercise program on muscle strength, resting metabolic rate, and body composition in individuals following bariatric surgery.

## Methods

### Participants

The sample size was calculated using G*Power software (version 3.1.9). A minimum of 10 participants per group was planned. Accordingly, 10 participants were assigned to HEG and 10 to CG, ensuring homogeneity with respect to age, BMI, and body fat percentage. Specifically, the study enrolled a total of 20 adult participants (15 females, 5 males) aged 19–52 years, all of whom underwent bariatric surgery using the sleeve gastrectomy (SG) procedure at the Department of General Surgery, Faculty of Medicine, a state university, with a BMI ≥ 30 kg/m^2^.

Eligibility criteria included absence of participation in any other exercise program, and no physical or mental impairments that could prevent participation in the study for both the exercise and control groups. Participation was voluntary, and all participants provided written informed consent prior to inclusion. No financial compensation was provided to any participant in either group.

Ethical approval was obtained from the Marmara University Clinical Research Ethics Committee (Protocol No: 09.2021.803). Additionally, the study received funding support from the Marmara University Scientific Research Projects Commission on December 24, 2021 (Project ID: 10382).

### Assessments

Participants were invited for baseline assessments three months post-sleeve gastrectomy (SG). Demographic information and body composition measurements—including body mass index (BMI), body fat percentage, fat mass, and lean body mass—were collected. Anthropometric assessments included body weight, height, waist circumference, and hip circumference. Resting metabolic rate and muscle strength were also evaluated. Additionally, participants underwent a cardiopulmonary exercise test (CPET) and completed the International Physical Activity Questionnaire (IPAQ) to assess physical activity levels. Following the four-month exercise intervention, all participants were invited to complete post-intervention assessments using the same measures to evaluate changes in body composition, metabolic rate, muscle strength, and physical activity levels.

#### Anthropometrics and Body Composition

Anthropometric measurements, including body weight, height, waist circumference, and hip circumference, were performed according to the standardized techniques recommended by the International Society for the Advancement of Kinanthropometry (ISAK) [25]. Height was measured barefoot using a stadiometer (SECA, Germany) with 0.01 m precision, while body weight and composition were assessed using a bioelectrical impedance analyzer (TANITA Body Composition Analyzer TBF-300, Corp., Tokyo, Japan) with 0.1 kg precision. Body composition measurements were conducted in the morning at 08:30 following a 12-h fast, and participants were instructed to avoid any food, beverages, or stimulants (e.g., caffeine-containing drinks) during this period. Body composition outcomes included BMI, body fat percentage (%), fat mass (kg), and lean body mass (kg). Obesity classification followed World Health Organization criteria: BMI ≥ 30 kg/m^2^ (Class I obesity), ≥ 35 kg/m^2^ (Class II obesity), and ≥ 40 kg/m^2^ (Class III obesity) [26].

#### Resting Metabolic Rate

Resting metabolic rate (RMR) was measured via indirect calorimetry using the QUARK-RMR system (COSMED, Italy). Participants fasted for at least 12 h before testing and abstained from alcohol, nicotine, caffeine, and strenuous physical activity. Female participants were not assessed during the menstrual phase of their cycle; testing sessions were scheduled outside the menstruation period. Measurements were conducted in a quiet, dimly lit room maintained at 20–25 °C. The first 5 min of the 15-min measurement were allocated for stabilization, and the mean of the final 10 min was automatically calculated by the device. Resting oxygen uptake (VO_₂_) was converted to kcal/day using the Weir equation: kcal/day = [(3.941 × VO_₂_) + (1.106 × VCO_₂)_] × 1440 [27].

#### Muscle Strength

Isometric muscle strength was assessed by a certified exercise specialist using the Lafayette Manual Muscle Tester (MMT). This method has been validated as reliable for clinical muscle strength evaluation compared to isokinetic devices [28, 29]. Participants performed a brief familiarization trial, followed by a maximal contraction sustained for 5 s, with a 1-min rest interval. Measurements were conducted using the “break test” method [28] and standardized positions as defined by Kendall and McCreary [30]. Upper limb assessments included elbow and shoulder flexion/extension, and lower limb assessments included hip and knee flexion/extension, performed bilaterally. Handgrip strength was assessed using a digital hand dynamometer (Takei Scientific Instruments Co.,Ltd., Japan), with measurements performed bilaterally; two trials were conducted for each hand, and the highest value was recorded for analysis. SMI (Skeletal Muscle Index) was calculated as appendicular skeletal muscle mass (ASM, kg) divided by height squared (m²) (SMI = ASM/ height²). Sex-specific cut-off values for low muscle mass and strength (SMI <7.0 kg/m² for men and <5.5 kg/m² for women; handgrip strength<27 kg for men and <16 kg for women) were implemented in accordance with the consensus criteria proposed by the European Working Groupon Sarcopenia in Older People [31].

#### Cardiopulmonary Capacity

Cardiopulmonary capacity was assessed via a graded treadmill exercise test (Tepa, TM-PRO 2000, Cosmed) using a customized ramp protocol adapted from the Bruce protocol. The test began at 2.7 km/h and 10% incline, with incremental increases in speed and incline every 3 min across eight stages, reaching a final workload of 8.8 km/h at 20% incline. This protocol emphasizes incline rather than speed, suitable for individuals with obesity. Participants fasted for at least 2 h before testing and avoided caffeinated or sugary beverages. Continuous 12-lead ECG monitoring, arterial blood pressure measurements, and secure oronasal mask fitting (Hans Rudolph, USA) ensured safety. The test concluded upon volitional fatigue, arrhythmia onset, VO2 plato or participant request, and VO_₂_peak was determined breath-by-breath using the Metalyzer 3B system (Cortex, Germany). The cardiopulmonary exercise test was performed at baseline and post-intervention under physician supervision.

#### Hybrid-Type Multicomponent Exercise Program

The hybrid exercise program was administered under the supervision of a certified exercise specialist, delivered both online and in-person. Training sessions were scheduled on non-consecutive days, three times per week (Monday, Wednesday, Friday), with make-up sessions available on Tuesday, Thursday, and Saturday in case of missed sessions. Prior to commencing the program, participants underwent a one-week adaptation period to familiarize them with correct exercise techniques, equipment setup, and safety procedures, supported by practical instruction.

**Fig. 1:**
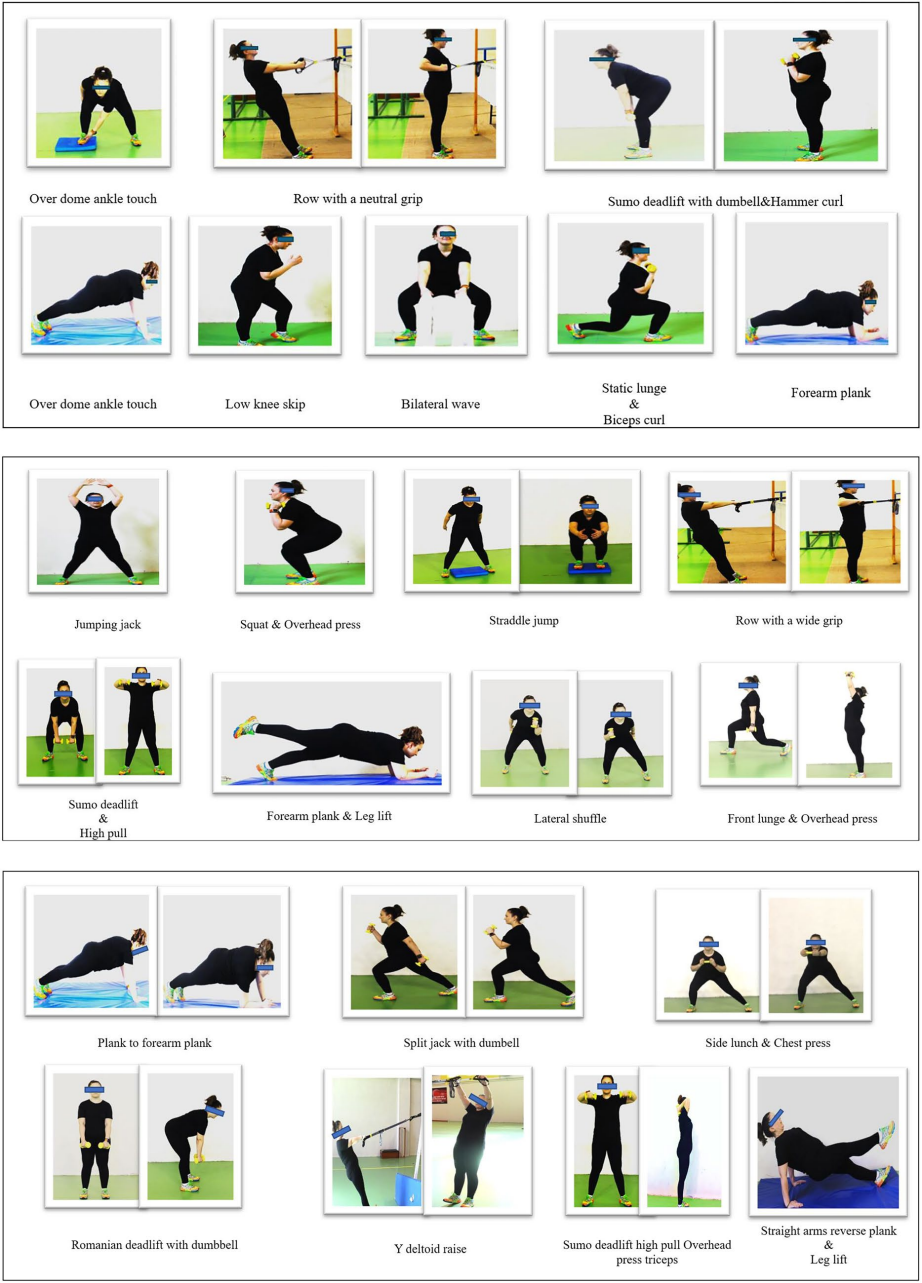

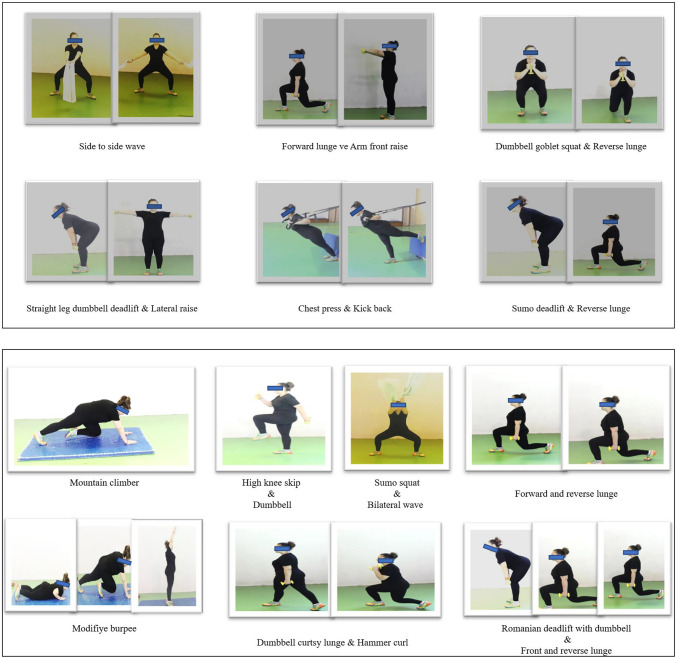
Visual explanation of the applied hybrid exercise program

The program was developed in accordance with the American College of Sports Medicine’s guidelines for individuals with obesity and consisted of four progressive phases over 16 weeks, totaling 48 sessions [32–34]. Tables 1 and 2 show that each phase included gradual increases in exercise duration and intensity: Phase 1 lasted approximately 53 min per session (159 min/week), Phase 2 lasted 56 min per session (168 min/week), and Phases 3 and 4 lasted approximately 71 min per session (213 min/week). Free weights were employed, and individual load intensity was determined using the 10RM method, considered safe for individuals with obesity [35, 36]. Upper limb training included biceps curls, while lower limb exercises included front squats. (Table 1, Table 2; Fig. 1).

**Table 1:** Overview of the hybrid exercise model

Phases Weeks	Phase 1	Phase 2	Phase 3	Phase 4
	1–4	5–8	9–12	13–16
Number of Exercises	10	11	11	11
Duration of Effort (s)	20	30	40	40
Rest Between Exercises (s)	40	30	20	20
Training Frequency	½	1/1	2/1	2/1
Rest Between Sets (min)	4	4	4	4
Number of Sets	3	3	4	4
Session Duration (min)	~ 38	~ 41	~ 56	~ 56
Effort-to-Session Ratio (%)	%26,3	%40,2	% 52,3	%52,3
Number of Repetitions	Execution of movement patterns with proper technique and maximal speed

**Table 2:** Progression of the hybrid exercise intervention program

Phases Weeks	Phase 1	Phase 2	Phase 3	Phase 4
	1–4	5–8	9–12	13–16
Movement 1	Over dome ankle touch	Straddle jump	Split jack	Straddle jump
Movement 2	Row with a neutral grip	Row with a wide grip	Y deltoid raise	Chest press&Kick back
Movement 3	Sumo deadlift with dumbbell&Hammer curl	Sumo deadlift&High pull	Sumo deadlift& Overhead press and triceps	Sumo deadlift&Reverse lunge
Movement 4	Plank with straight arms	Forearm plank&Leg lift	Straight arms reverse plank&Leg lift	Mountain climber
Movement 5	Low knee skip	Lateral shuffle	Jumping jack with dumbbell	High knee skip with dumbbell
Movement 6	Bilateral wave	Alternating wave	Side-to-side wave	Sumo squat&Bilateral wave
Movement 7	Static lunge&Biceps curl	Front lunge overhead press	Forward lunge&Arm front raise	Forward and reverse lunge&Arm front raise
Movement 8	Forearm plank	Plank to forearm plank	Shifting Plank	Forearm plank&Leg lift
Movement 9	Jumping jack	Split jack with dumbell	Low knee skip with dumbbell	Modifiye burpee
Movement 10	Squat&Overhead press	Side lunch&Chest press	Dumbbell goblet squat&Reverse lunge	Dumbblee curtsy lunge&Hammer curl
Movement 11		Romanian deadlift with dumbbell	Straight-Leg Dumbbell Deadlift&Lateral raise	Romanian deadlift with dumbbell&Front and reverse lunge

Standardized instructions were provided to minimize measurement errors, and movements were demonstrated by the specialist prior to participant execution. Participants received verbal encouragement, and weights were precisely calibrated. Rest intervals of 3–5 min were provided between sets. Repetition volume was adjusted according to participants’ feedback and technical proficiency. Exercise intensity was subjectively assessed using the Borg Rating of Perceived Exertion (RPE) scale (6–20), with categories defined as “10–11: light,” “12–13: somewhat hard,” and “14–15: hard” [37]. Objective monitoring of exercise intensity was conducted using target heart rate zones calculated via the Karvonen formula, with continuous tracking provided by telemetric heart rate monitors (Kalenji 10 Rhythm 110). The devices emitted audible alerts when participants exceeded pre-defined heart rate zones, ensuring precise regulation of exercise intensity throughout the sessions.

### Statistical Analysis

Descriptives presented with mean (SD, standard deviation) when data met parametric test assumptions, otherwise reported as median (IQR, interquartile range) for numeric variables. Descriptives of categorical variables presented with frequency (percent). Study groups compared using independent samples T-test, Fisher’s Exact test and Fisher-Freeman-Halton test. Pre and post intervention compared with respect to study group and sex with three-way Repeated Measures ANOVA (analysis of variance) when parametric assumptions met, otherwise Rank Aligned Transform ANOVA applied [38]. Posthoc pairwise comparisons were reported with Bonferroni adjustment. The mean percent change from pre- to post-intervention reported with 95% confidence interval. Effect sizes were reported using Cohen’s d (small: 0.2–0.5; medium: 0.5–0.8; large: ≥ 0.8) and partial eta squared (η^2^p; small: 0.01–0.06; medium: 0.06–0.14; large: ≥ 0.14) [39, 40]. Statistical significance set at p < 0.05 level. Statistical software JASP [41], and R 4.2.3 packages ARTool [42, 43], rstatix [44] and effectsize were utilized for statistical analysis [45].

## Results

### Participant Characteristics

The study groups had similar sex distribution (female: n=8 in the HEG vs n=7 in the CG; male: n=2 vs n=3, respectively) (p > 0.05). The mean age was 34.7 (11.39) years ranging between 19–52 years in the HEG, and 35.2 (9.30) years ranging between 25–50 years in the CG (*p* > 0.05). All participants had social security and were living in city. All the participants had hypertension and blood glucose level below 110. No other chronic disease was present, also none of the participants had a relative with cardiovascular disease or sudden death. None of the participants had regular exercise before the intervention (Table 7). The study groups had similar marital status, alcohol consumption habit, smoking habit, education level, employment status, income level, housing condition, home ownership status, and walking aid need (p > 0.05) (Table 3).

**Table 3:** Characteristics of study participants

Characteristic		HEG		CG		*p*
		*n* (%)	Mean (SD)	*n* (%)	Mean (SD)	
Sex	Male	2 (20)		3 (30)		1.000ᵃ
	Female	8 (80)		7 (70)		
Age		10	34.70 (11.39)	10	35.20 (9.30)	0.916ᵇ
Marital Status	Single	5 (50)		1 (10)		0.141ᵃ
	Married	5 (50)		9 (90)		
Alcohol Consumption	Absent	7 (70)		7 (70)		1.000ᵃ
	Rarely	3 (30)		3 (30)		
Smoking	Present	6 (60)		6 (60)		1.000ᵃ
	Absent	4 (40)		4 (40)		
Education Level	High school	5 (50)		4 (40)		0.656ᶜ
	Undergraduate	4 (40)		6 (60)		
	Postgraduate	1 (10)		0 (0)		
Employment	Unemployed	4 (40)		4 (40)		0.637ᶜ
	Office job	1 (10)		3 (30)		
	Physically active job	5 (50)		3 (30)		
Income Level	< Minimum wage	4 (40)		4 (40)		0.227ᶜ
	Minimum wage	4 (40)		1 (10)		
	2 × Minimum wage	2 (20)		2 (20)		
	3 × Minimum wage	0 (0)		3 (30)		
Housing	Apartment	9 (90)		8 (80)		1.000ᵃ
	Standalone house	1 (10)		2 (20)		
Home ownership	Self	2 (20)		4 (40)		0.848ᶜ
	Family	5 (50)		3 (30)		
	Rent	3 (30)		3 (30)		
Walking aid	Present	1 (10)		0 (0)		1.000ᵃ
	Absent	9 (90)		10 (100)		

### Anthropometrics and Body Composition

Table 4 presents the anthropometric and body composition characteristics of HEG and CG at baseline and after the 16-week intervention period. A significant main effect of time was observed for body weight, BMI, waist and hip circumference, body fat percentage, fat mass, lean body mass, and total body water (*p* < 0.001), indicating overall changes throughout the intervention period.

**Table 4:** Anthropometric and body composition characteristics of study groups at baseline and after 16 weeks of intervention period

Measure	Sex	HEG			CG			Effect (*p*; partial eta squared)						
		Pre-int	Post-int	Mean % change (95% CI)	Pre-int	Post-int	Mean % change (95% CI)	T	G	S	TxG	TxS	GxS	TxGxS
Body Mass (kg)	M	99.93 (6.46)	85.3 (8.52)	−14.59 (−23.58 - 3.41)	96.35 (6.58)	86.8 (3.54)	−9.58 (−98 - 78.84)	< 0.001*; 0.717	0.286; 0.071	0.052; 0.216	0.109; 0.153	0.878; 0.002	0.206; 0.098	0.747; 0.007
	F	83.61 (7.16)	68.34 (11.73)	−18.56 (−27.52 - −9.60)	91.64 (9.91)	83.89 (12.23)	−8.6 (−14.33 - −2.87)							
	Total	88.51 (10.28)	73.43 (13.23)	−17.37 (−23.68 - −11.05)	92.58 (9.22)	84.47 (10.92)	−8.8 (−13.73 - −3.87)							
Waist Circumference (cm)	M	106.33 (10.41)	91.83 (5.48)	−13.43 (−21.36 - −5.49)	102.75 (2.47)	94.5 (14.85)	−8.18 (−118.15 - 101.8)	< 0.001*; 0.799	0.087; 0.172	0.187; 0.106	0.019*; 0.3	0.367; 0.051	0.073; 0.187	0.865; 0.002
	F	88.71 (8.55)	76.14 (11.28)	−14.51 (−19.47 - −9.56)	104.06 (9.74)	98.63 (10.61)	−5.29 (−8.34 - −2.24)							
	Total	94 (12.05)	80.85 (12.2)	−14.19 (−17.52 - −10.86)	103.8 (8.65)	97.8 (10.73)	−5.87 (−9.68 - −2.05)							
Hip Circumference (cm)	M	106.07 (3.1)	98.67 (4.16)	−7 (−10.92 - −3.08)	110.25 (9.55)	101 (2.12)	−7.96 (−96.85 - 80.93)	< 0.001*; 0.722	0.083; 0.176	0.016*; 0.311	0.100; 0.16	0.319; 0.062	0.527; 0.026	0.032*; 0.257
	F	116.79 (2.34)	99 (8.03)	−15.27 (−20.86 - −9.67)	117.25 (5.31)	112.06 (7.17)	−4.45 (−7.32 - −1.58)							
	Total	113.57 (5.71)	98.9 (6.84)	−12.79 (−17.36 - −8.21)	115.85 (6.39)	109.85 (7.89)	−5.15 (−8.53 −1.78)							
Waist-to-Hip Ratio (WHR)	M	1 (0.08)	0.93 (0.05)	−6.9 (−16.01 - 2.21)	0.94 (0.1)	0.93 (0.13)	−0.37 (−23.64 - 22.9)	0.100; 0.16	0.226; 0.09	0.002*; 0.456	0.218; 0.093	0.100; 0.16	0.047*; 0.224	0.063; 0.20
	F	0.76 (0.07)	0.77 (0.08)	0.97 (−1.51 - 3.46)	0.89 (0.05)	0.88 (0.06)	−0.75 (−5.4 - 3.9)							
	Total	0.83 (0.14)	0.82 (0.1)	−1.39 (−4.77 - 1.98)	0.9 (0.06)	0.89 (0.07)	−0.68 (−4.24- 2.89)							
BMI (kg/m2)	M	31.4 (1.04)	26.87 (2.06)	−14.38 (−31.4 - 2.63)	33.75 (2.33)	30.4 (1.27)	−9.58 (−99.63 - 80.47)	< 0.001*; 0.633	0.086; 0.173	0.384; 0.048	0.154; 0.123	0.621; 0.016	0.846; 0.002	0.445; 0.037
	F	33.73 (3.07)	27.06 (2.42)	−19.22 (−28.54 - −9.89)	35.49 (4.58)	32.6 (5.36)	−8.28 (−14.34 - −2.23)							
	Total	33.03 (2.79)	27 (2.2)	−17.77 (−24.31 - −11.23)	35.14 (4.18)	32.16 (4.83)	−8.54 (−13.72—−3.37)							
BMR (kcal)	M	1830.33 (398.2)	1877.33 (110.46)	4.75 (−32.9 - 42.4)	2223.5 (211.42)	1830.33 (398.2)	−15.49 (−169.41 - 138.43)							
	F	1546.57 (282.99)	1161.29 (469.06)	−26.26 (−42.6 - −9.91)	1561.88 (316.65)	1546.57 (282.99)	−15.2 (−24.23 - 6.17)	0.003*; 0.443	0.416; 0.042	0.006*; 0.388	0.357; 0.053	0.277; 0.073	0.794; 0.004	0.041*; 0.235
	Total	1631.7 (327.74)	1376.1 (518.68)	−16.95 (−32.69 - −1.22)	1694.2 (400.97)	1631.7 (327.74)	−15.26 (−23.2 - −7.31)							
Fat Percentage (%)	M	21.77 (5.17)	17.47 (4.1)	−19.67 (−26.45 - −12.88)	22.35 (2.19)	17.2 (3.96)	−21.8 (−249.89 - 206.3)	0.001*; 0.491	0.142; 0.13	< 0.001*; 0.786	0.291; 0.069	0.306; 0.065	0.159; 0.12	0.195; 0.102
	F	39.9 (3.66)	27.67 (7.13)	−29.82 (−48.04 - −11.59)	42.8 (4.25)	38.61 (6.59)	−9.97 (−18.49 - −1.45)							
	Total	34.46 (9.57)	24.61 (7.87)	−26.77 (−38.84 - −14.71)	38.71 (9.43)	34.33 (10.82)	−12.34 (−21.86 - −2.81)							
Fat Mass (kg)	M	22.07 (6.47)	15.03 (4.5)	−31.37 (−52.48 - −10.26)	21.6 (3.54)	15 (4.1)	−28.04 (−304.46 - 248.39)	< 0.001*; 0.612	0.204; 0.099	0.002*; 0.454	0.265; 0.077	0.342; 0.056	0.182; 0.108	0.320; 0.062
	F	33.47 (3.94)	19.69 (7.55)	−40.91 (−61.22 - −20.6)	39.5 (7.89)	33.06 (10.59)	−17.09 (−29.69 - −4.5)							
	Total	30.05 (7.07)	18.29 (6.89)	−38.05 (−51.6 - −24.5)	35.92 (10.33)	29.45 (12.13)	−19.28 (−31.73 - −6.83)							
Lean Body Mass (kg)	M	74.13 (7.4)	70.27 (5.88)	−5.08 (−12.62 - 2.46)	74.75 (3.04)	71.8 (0.57)	−3.88 (−32.21 - 24.45)	< 0.001*; 0.7	0.523; 0.026	< 0.001*; 0.841	0.417; 0.042	0.017*; 0.307	0.837; 0.003	0.734; 0.007
	F	50.2 (5.79)	48.67 (5.45)	−3.01(−4.5 - −1.51)	52.1 (3.37)	50.95 (2.77)	−2.13 (−4.14 - -.012)							
	Total	57.38 (12.97)	55.15 (11.67)	−3.63 (−5.2 - −2.06)	56.63 (10.05)	55.12 (9.13)	−2.48 (−4.26 - −0.71)							
Total Body Water (kg)	M	54.27 (5.44)	51.47 (4.31)	−5.02 (−12.59 - 2.55)	54.75 (2.19)	52.55 (0.35)	−3.95 (−32.7 - 24.79)	< 0.001*; 0.72	0.530; 0.025	< 0.001*; 0.841	0.505; 0.028	0.016*; 0.31	0.844; 0.002	0.688; 0.01
	F	36.74 (4.24)	35.64 (3.97)	−2.95 (−4.48 - −1.43)	38.16 (2.49)	37.21 (2.01)	−2.41 (−4.14 −0.69)							
	Total	42 (9.5)	40.39 (8.55)	−3.57 (−5.15 - −1.99)	41.48 (7.37)	40.28 (6.71)	−2.72 (−4.3 - −1.14)							
Right Leg Fat Percentage (%)	M	15.9 (4.77)	13.77 (3.97)	−13.15 (−17.05 - −9.25)	14.45 (2.76)	11.15 (5.73)	−25.26 (−253.23 - 202.71)	0.002*; 0.449	0.433; 0.039	< 0.001*; 0.886	0.311; 0.064	0.130; 0.137	0.117; 0.146	0.159; 0.12
	F	42.69 (3.37)	32.27 (5.66)	−23.73 (−38.17 - −9.29)	45.16 (4.86)	41.55 (6.87)	−8.32 (−14.9 - −1.74)							
	Total	34.65 (13.42)	26.72 (10.24)	−20.55 (−30.39 - −10.71)	39.02 (13.67)	35.47 (14.31)	−11.7 (−21.05 - −2.36)							
Right Leg Fat Mass (kg)	M	2.5 (0.75)	1.97 (0.61)	−21.55 (−26.93 - −16.16)	2.45 (0.35)	1.7 (0.85)	−32.41 (−255.91 - 191.13)	< 0.001*; 0.549	0.296; 0.068	< 0.001*; 0.69	0.343; 0.056	0.054; 0.212	0.208; 0.097	0.189; 0.105
	F	6.64 (0.74)	4.21 (1.23)	−35.97 (−53.69 - −18.26)	7.64 (1.71)	6.5 (1.99)	−15.55 (−26.32 - −4.77)							
	Total	5.4 (2.12)	3.54 (1.51)	−31.65 (−43.92 - −19.37)	6.6 (2.66)	5.54 (2.69)	−18.92 (−30.2 - −7.64)							
Right Leg Lean Mass (kg) ᵃ	M	14 (10.9–14.4)	13 (10.7–13.1)	−4.13 (−33.32 - 25.05)	14.05 (12.8–15.3)	13.45 (12.7–14.2)	−3.9 (−44.7 - 36.72)	< 0.001*; 0.72	0.869; 0.001	< 0.001*; 0.57	0.056; 0.24	0.009*; 0.37	0.834; 0.002	0.250; 0.08
	F	8.7 (8.1–9.9)	8.3 (7.9–9.5)	−3.54 (−4.78 - −2.29)	9.1 (8.55–9.8)	8.95 (8.5–9.35)	−2.26 (−3.8 - −0.72)							
	Total	9.35 (8.4–10.9)	8.9 (8.1–10.7)	−3.67 (−4.94 - −2.39)	9.5 (8.8–9.9)	9.15 (8.8–9.7)	−2.61 (−4.28 - −0.93)							
Left Leg Fat Percentage (%)	M	16.43 (5.41)	14.57 (4.48)	−10.76 (−20.23 - −1.3)	15.8 (1.7)	13.1 (4.67)	−18.2 (−204.65 - 168.24)	0.004*; 0.418	0.368; 0.051	< 0.001*; 0.882	0.293; 0.069	0.110; 0.152	0.189; 0.105	0.181; 0.109
	F	43.11 (2.91)	32.94 (5.68)	−23.07 (−37.1 - −9.03)	45.19 (4.86)	41.7 (6.77)	−7.93 (−15.11 - −0.74)							
	Total	35.11 (13.35)	27.43 (10.23)	−19.38 (−29.29 - −9.46)	39.31 (13.12)	35.98 (13.55)	−9.98 (−17.95 - −2.01)							
Left Leg Fat Mass (kg)	M	2.5 (0.85)	2.03 (0.67)	−18.45 (−25.72 - −11.18)	2.55 (0.07)	1.9 (0.71)	−25.85 (−256.51 - 204.82)	< 0.001*; 0.516	0.274; 0.074	< 0.001*; 0.676	0.322; 0.061	0.051; 0.217	0.250; 0.082	0.194; 0.103
	F	6.57 (0.67)	4.2 (1.27)	−35.71 (−53.44 - −17.97)	7.5 (1.69)	6.44 (1.98)	−14.74 (−26 - −3.49)							
	Total	5.35 (2.08)	3.55 (1.5)	−30.53 (−43.26 - −17.8)	6.51 (2.57)	5.53 (2.6)	−16.96 (−27.96 - −5.97)							
Left Leg Lean Mass (kg) ᵃ	M	13.4 (10.7–14.1)	12.6 (10.3–12.6)	−6.78 (−15.53 - 1.96)	13.45 (12.4–14.5)	12.65 (12.2–13.1)	−5.63 (−56.73 - 45.46)	< 0.001*; 0.7	0.871; 0.001	< 0.001*; 0.58	0.361; 0.1	0.007*; 0.38	0.978; 0.001	0.769; 0.001
	F	8.4 (8–9.6)	8.2 (7.6–9.2)	−4.16 (−5.63 - −2.69)	8.9 (8.5–9.5)	8.7 (8.35–9.05)	−2.81 (−4.86 - −0.76)							
	Total	9.1 (8.3–10.7)	8.75 (7.8–10.3)	−4.94 (−6.7 - −3.19)	9.2 (8.7–9.8)	8.8 (8.5–9.3)	−3.38 (−5.6 - −1.15)							
Right Arm Fat Percentage (%)	M	21.5 (3.73)	19.7 (4.1)	−8.71 (−16.34 - −1.07)	24.3 (4.24)	19.85 (1.48)	−16.51 (−202.38 - 169.37)	0.002*; 0.457	0.104; 0.157	< 0.001*; 0.844	0.415; 0.042	0.096; 0.163	0.291; 0.069	0.120; 0.144
	F	46.24 (4.54)	33.23 (5.72)	−27.13 (−42.2 - −12.05)	48.74 (5.2)	43.88 (7.13)	−10.11 (−17.85 - −2.37)							
	Total	38.82 (12.64)	29.17 (8.26)	−21.6 (−33.1 - −10.1)	43.85 (11.37)	39.07 (11.93)	−11.39 (−19.28 - −3.51)							
Right Arm Fat Mass (kg)	M	1.17 (0.31)	1 (0.26)	−14.21 (−23.19 - −5.23)	1.35 (0.35)	1 (0.14)	−21.88 (−299.82 - 256.07)	< 0.001*; 0.564	0.199; 0.101	0.007*; 0.375	0.468; 0.033	0.057; 0.209	0.332; 0.059	0.122; 0.143
	F	2.11 (0.29)	1.19 (0.39)	−43.19 (−60.81 - −25.57)	2.5 (0.71)	2.06 (0.78)	−18.17 (−31.38 - −4.96)							
	Total	1.83 (0.54)	1.13 (0.35)	−34.5 (−49.52 - −19.47)	2.27 (0.8)	1.85 (0.82)	−18.91 (−31.36 - −6.46)							
Right Arm Lean Mass (kg)	M	4.2 (0.35)	3.87 (0.15)	−7.7 (−18.95 - 3.56)	4.3 (0.14)	4.1 (0)	−4.6 (−32.79 - 23.59)	< 0.001*; 0.696	0.249; 0.082	< 0.001*; 0.895	0.090; 0.169	0.008*; 0.363	0.987; 0.001	0.648; 0.013
	F	2.44 (0.37)	2.31 (0.32)	−5.03 (−9.16 - −0.90)	2.58 (0.21)	2.53 (0.23)	−2 (−3.8 - −0.2)							
	Total	2.97 (0.92)	2.78 (0.8)	−5.83 (−8.99 - −2.67)	2.92 (0.75)	2.84 (0.69)	−2.52 (−4.26 - −0.78)							
Left Arm Fat Percentage (%)	M	21.97 (2.63)	19.07 (2.06)	−13.1 (−18.44 - −7.76)	25.1 (4.95)	20.55 (0.64)	−16.25 (−187.42 - 154.92)	< 0.001*; 0.508	0.087; 0.172	< 0.001*; 0.844	0.345; 0.056	0.124; 0.142	0.412; 0.043	0.150; 0.125
	F	46.64 (5.06)	34.16 (5.2)	−25.87 (−39.39 - −12.35)	49.11 (5.05)	44.23 (7.28)	−10.15 (−17.89 - −2.4)							
	Total	39.24 (12.68)	29.63 (8.49)	−22.04 (−31.68 - −12.4)	44.31 (11.18)	39.49 (11.87)	−11.37 (−18.99 - −3.74)							
Left Arm Fat Mass (kg)	M	1.2 (0.3)	0.97 (0.21)	−18.7 (−36.18 - −1.23)	1.45 (0.35)	1.05 (0.07)	−24.76 (−233.41 - 183.9)	< 0.001*; 0.589	0.188; 0.106	0.009*; 0.356	0.501; 0.029	0.070; 0.191	0.404; 0.044	0.170; 0.114
	F	2.26 (0.35)	1.27 (0.41)	−42.73 (−60.41 - −25.05)	2.74 (0.87)	2.23 (0.93)	−19.5 (−32.94 - −6.06)							
	Total	1.94 (0.6)	1.18 (0.38)	−35.52 (−49.64 - −21.41)	2.48 (0.95)	1.99 (0.96)	−20.55 (−32.22 - −8.89)							
Left Arm Lean Mass (kg)	M	4.3 (0.46)	4.03 (0.35)	−6.04 (−14.09 - 2.02)	4.35 (0.07)	4.1 (0.14)	−5.71 (−48.69 - 32.27)	< 0.001*; 0.724	0.382; 0.048	< 0.001*; 0.848	0.331; 0.059	0.203; 0.099	0.584; 0.019	0.464; 0.034
	F	2.57 (0.39)	2.34 (0.37)	−8.95 (−13.21 - −4.69)	2.76 (0.28)	2.65 (0.28)	−4.07 (−7.6 - −0.54)							
	Total	3.09 (0.92)	2.85 (0.89)	−8.08 (−11.15 - −5)	3.08 (0.71)	2.94 (0.66)	−4.4 (−7.34 - −1.46)							
Trunk Fat Percentage (%)	M	23.97 (5.12)	19.03 (4.54)	−20.91 (−26.76 - −15.06)	26.35 (4.45)	19.8 (3.68)	−22.57 (−265.56 - 220.41)	0.002*; 0.468	0.066; 0.196	< 0.001*; 0.587	0.339; 0.057	0.410; 0.043	0.208; 0.097	0.182; 0.108
	F	36.63 (4.16)	22.84 (9.02)	−36.47 (−60.49 - −12.45)	39.76 (3.93)	35.38 (6.39)	−11.07 (−21.35 - −0.80)							
	Total	32.83 (7.4)	21.7 (7.89)	−31.8 (−47.91 - −15.68)	37.08 (6.8)	32.26 (8.74)	−13.37 (−24.04 - −2.71)							
Trunk Fat Mass (kg)	M	12.27 (3.74)	9.13 (2.95)	−25.83 (−44.92 - −6.75)	14.1 (3.39)	9.3 (2.4)	−29.96 (−334.63 - 274.71)	< 0.001*; 0.578	0.091; 0.168	0.049*; 0.221	0.559; 0.022	0.513; 0.027	0.241; 0.085	0.169; 0.115
	F	15.93 (2.17)	8.63 (4.52)	−45.88 (−70.86 - −20.91)	19.14 (3.24)	15.86 (5.01)	−17.73 (−31.88 - −3.59)							
	Total	14.83 (3.06)	8.78 (3.95)	−39.87 (−57.29 - −22.45)	18.13 (3.73)	14.55 (5.27)	−20.18 (−34.07 - −6.29)							
Trunk Lean Mass (kg)	M	39.77 (3.11)	38.3 (2.92)	−3.65 (−10.61 - 3.18)	38.65 (0.35)	37.5 (0.99)	−2.98 (−18.02 - 12.06)	< 0.001*; 0.573	0.969; 0.001	< 0.001*; 0.803	0.790; 0.005	0.091; 0.168	0.456; 0.035	0.619; 0.016
	F	27.61 (3.46)	27.09 (3.16)	−1.81 (−4.01 - 0.38)	28.73 (1.82)	28.1 (1.48)	−2.1 (−4.06 - −0.14)							
	Total	31.26 (6.68)	30.45 (6.16)	−2.36 (−4.16 - −0.57)	30.71 (4.48)	29.98 (4.19)	−2.28 (−3.83 - −0.72)							

Abbreviations: *HEG* hybrid exercise group, *CG* control group, *Pre-int* pre-intervention, *Post-int* post-intervention, *M* male, *F* female, *T *time, *G* group, *S* sex, *CI* confidence interval. Descriptives shown with Mean(SD) for three-way repeated measure ANOVA. Statistically significant difference at *p < 0.05 level

Body weight and BMI decreased significantly over time in both groups, with relatively greater reductions observed in the HEG. Similarly, waist and hip circumferences showed significant time-dependent decreases in both groups, with more pronounced reductions in the HEG compared to the CG. A significant main effect of sex was detected for the waist-to-hip ratio; however, no significant time × group interaction was observed.

Regarding body composition, body fat percentage and fat mass decreased significantly over time in both groups, with greater reductions in the HEG compared to the CG. These decreases were consistently observed across all regional measurements, including the trunk, upper extremities (right and left arms), and lower extremities (right and left legs). Significant main effects of time were identified for regional fat parameters (*p* < 0.01), whereas group interactions were mostly non-significant. Lean body mass and total body water exhibited significant time effects, with modest reductions observed in both groups during the intervention period. Males demonstrated significantly higher lean body mass and total body water values than females at both time points; however, no significant time × group × sex interactions were detected for these parameters. Trunk fat percentage and trunk fat mass decreased significantly over time in both groups, with greater absolute reductions observed in the HEG. In contrast, trunk lean mass showed a small but significant decrease over time, independent of group allocation.

### Muscle Strength

Table 5 presents the effects of the hybrid exercise intervention on upper- and lower-body strength, handgrip strength, and skeletal muscle index (SMI), stratified by group and sex. Significant time × group interactions were observed for all upper- and lower-extremity strength parameters (right and left upper-body strength and right and left lower-body strength; *p* < 0.001), with large effect sizes (partial η^2^ = 0.64–0.93). In the HEG, both male and female participants demonstrated significant increases in upper- and lower-body strength following the intervention, whereas changes in the CG were smaller or non-significant. Across both assessment points, males exhibited higher absolute strength values than females. Handgrip strength demonstrated a significant main effect of time and a significant time × group interaction, with increases observed in the HEG (p < 0.001), while no significant changes were detected in the CG. SMI decreased significantly over time in both groups. No significant time × group × sex interaction was observed, indicating that changes in SMI were comparable between males and females across groups.

**Table 5:** Strength characteristics of study groups at baseline and after 16 weeks of intervention period

Measure	Sex	HEG			CG			Effect (*p*; partial eta squared)						
		Pre-int	Post-int	Mean % change (95% CI)	Pre-int	Post-int	Mean % change (95% CI)	T	G	S	TxG	TxS	GxS	TxGxS
RUB-S (*N*) ᵃ	M	427 (421.1–431.8)	684.8 (677.8–688.3)	60.25 (58.29 - 62.2)	448.5 (434.7–462.3)	523.6 (507–540.2)	16.74 (15.35 - 18.13)	< 0.001*; 0.93	< 0.001*; 0.79	< 0.001*; 0.64	< 0.001*; 0.93	< 0.001*; 0.83	0.225; 0.09	0.725; 0.001
	F	306 (301.4–310.1)	503.5 (498.8–507.7)	64.94 (62.94 - 66.95)	305.95 (305.15–309.85)	333.55 (323–353.4)	9.48 (2.12 - 16.84)							
	Total	309.6 (301.9–421.1)	507.4 (501.2–677.8)	63.53 (61.46 - 65.61)	308.1 (305.4–311.1)	338.85 (330.3–372.6)	10.93 (4.96 - 16.9)							
LUB-S (*N*) ᵃ	M	412 (408.5–413.1)	662.6 (650.7–678)	61.42 (53.55 - 69.29)	425.5 (412.8–438.2)	512.6 (494.2–531)	20.45 (11.18 - 29.71)	< 0.001*; 0.93	< 0.001*; 0.8	< 0.001*; 0.64	< 0.001*; 0.93	< 0.001*; 0.84	0.246; 0.08	0.295; 0.07
	F	298.7 (289.5–299.4)	499.2 (498.1–508.7)	69.49 (67.46 - 71.51)	294.2 (292.75–299.6)	328.85 (314.5–339.8)	9.82 (1.43 - 18.2)							
RLB-S (*N*) ᵃ	Total	299.4 (292.1–408.5)	506.6 (498.3–650.7)	67.07 (63.82 - 30.31)	296.7 (293.1–305.2)	334.15 (316.2–362.6)	11.94 (4.84 - 19.04)							
	M	579.8 (568.2–601.7)	1049 (956.1–1050.1)	74.68 (50.19 - 99.17)	605.45 (603.8–607.1)	739 (738.7–739.3)	22.06 (17.2 - 26.92)	< 0.001*; 0.9	< 0.001*; 0.83	< 0.001*; 0.66	< 0.001*; 0.9	< 0.001*; 0.74	0.951; 0.001	0.105; 0.16
	F	383 (376.2–386.5)	761.6 (729.3–782.9)	98.22 (93.39 - 103.04)	384.8 (380.1–387.7)	461.6 (454.65–475.95)	19.88 (15.04 - 24.72)							
	Total	385.5 (379.5–568.2)	776 (744–956.1)	91.16 (81.86 - 100.46)	385.25 (381.7–393.4)	468.55 (457.3–488.3)	20.32 (16.6 - 24.03)							
LLB-S (*N*) ᵃ	M	590.9 (587.2–618)	1022.8 (1011.5–1027.3)	70.53 (60.55 - 80.51)	610.45 (609.6–611.3)	732.3 (724.1–740.5)	19.96 (0.77 - 39.15)	< 0.001*; 0.92	< 0.001*; 0.82	< 0.001*; 0.65	< 0.001*; 0.91	< 0.001*; 0.84	0.626; 0.02	0.799; 0.001
	F	378 (370–389.9)	733.9 (722.1–778.5)	96.83 (92.46 - 101.21)	378 (375.45–386.45)	445.05 (429.7–462.25)	17.05 (12.04 - 22.05)							
	Total	387.25 (370.6–587.2)	764.05 (728.6–1011.5)	88.94 (79.35 - 98.54)	382.15 (376.4–389.1)	454.45 (431.6–470.9)	17.63 (13.72 - 21.54)							
HG-R	M	34.5 (5)	44.6 (3.94)	30.01 (10.4 - 49.63)	38.45 (1.06)	40.85 (3.18)	6.17 (−41.87 - 54.21)	< 0.001*; 0.863	0.153; 0.123	< 0.001*; 0.8	< 0.001*; 0.815	0.029*; 0.265	0.139; 0.131	0.462; 0.034
	F	23.9 (3.28)	32.34 (1.86)	36.96 (23.77 - 50.15)	23.09 (4.25)	22.31 (3.62)	−2.54 (−10.35 - 5.26)							
	Total	27.08 (6.24)	36.02 (6.39)	34.88 (25.81 - 43.95)	26.16 (7.49)	26.02 (8.51)	−0.8 (−7.37 - 5.77)							
HG-L	M	35.43 (4.24)	43.7 (2.41)	24.08 (−0.35 - 48.51)	39.8 (1.41)	40.65 (0.64)	2.17 (−16.08 - 20.42)	< 0.001*; 0.79	0.259; 0.079	< 0.001*; 0.859	< 0.001*; 0.807	0.180; 0.11	0.131; 0.137	0.474; 0.033
	F	22.89 (2.56)	30.49 (1.82)	34.11 (24.67 - 43.54)	23.01 (4.15)	21.74 (3.42)	−4.81 (−12.68 - 3.06)							
	Total	26.65 (6.71)	34.45 (6.65)	31.1 (23.45 - 38.75)	26.37 (7.98)	25.52 (8.53)	−3.41 (−9.73 - 2.91)							
SMI (kg/m^2^)	M	10.85 (1.5)	10.1 (1.02)	−6.5 (−15.75 - 2.75)	12.66 (1.21)	12.01 (0.54)	−4.89 (−48.13 - 38.36)							
	F	8.98 (0.54)	8.57 (0.53)	−4.56 (−5.81 - −3.3)	9.06 (0.68)	8.81 (0.57)	−2.65 (−4.37 - −0.94)	< 0.001*; 0.745	0.017*; 0.306	< 0.001*; 0.739	0.404; 0.044	0.026*; 0.272	0.039*; 0.24	0.822; 0.003
	Total	9.54 (1.23)	9.03 (0.98)	−5.14 (−6.77 - −3.51)	9.78 (1.68)	9.45 (1.45)	−3.1 (−4.96 - −1.24)							

Abbreviations: *HEG* hybrid exercise group, *CG* control group, *Pre-int* pre-intervention, *Post-int* post-intervention, *M* male; *F* female, *T* time, *G* group, *S* sex, *CI* confidence interval. *RUB-S* right upper body strength, *LUB-S* left upper body strength, *RLB-S* right lower body strength, *LLB-S* left lower body strength, *N* newton, *HG-R* handgrip right, *HG-L* handgrip left, *SMI* appendicular skeletal muscle index; Descriptives shown with Mean(SD) for three-way repeated measure ANOVA or ᵃMedian(IQR) for Aligned Rank Transform ANOVA. Statistically significant difference at *p < 0.05 level

### Cardiopulmonary Capacity

Table 6 presents cardiopulmonary exercise test outcomes of HEG and CG at baseline and after the 16-week intervention period, stratified by sex. For relative peak oxygen uptake (VO₂peak, ml.kg⁻^1^.min⁻^1^), a significant main effect of time (*p* < 0.001; partial η^2^ = 0.788) and a significant time × group interaction (*p* < 0.001) were observed, indicating a greater improvement in the HEG compared with the CG. VO₂peak increased significantly in both male and female participants in the HEG, whereas no significant changes were detected in the CG. Absolute VO₂ (L.min⁻^1^) demonstrated a significant main effect of sex and a significant time × sex interaction (*p* < 0.05), with males exhibiting higher values than females at both assessment points. No significant time × group interaction was identified for this parameter. For percent predicted VO₂peak, a significant time × group interaction was observed (*p* = 0.003), characterized by an increase in the HEG and a decrease in the CG, particularly among female participants. The respiratory exchange ratio (RER) showed a significant main effect of time and a significant time × group interaction (*p* < 0.01). Minute ventilation (V̇E) demonstrated significant main effects of time and sex, along with a significant time × group interaction (*p* < 0.01), reflecting an enhanced ventilatory response in the HEG, while changes in the CG were limited. Among ventilatory pattern parameters, peak tidal volume (VTpeak) exhibited a significant time × group interaction (*p* < 0.05). Breathing frequency (BF) and breathing reserve (BR%) showed significant time × sex interactions. Regarding ventilatory efficiency indices, V̇E/V̇O₂ demonstrated significant main effects of time and group (*p* < 0.05), whereas no significant time × group interactions were observed for V̇E/V̇CO₂ or VD/VT.

**Table 6:** CPET data of study groups at baseline and after 16 weeks of intervention period

Measure	Sex	HEG			CG			Effect (*p*; partial eta squared)						
		Pre-int	Post-int	Mean % change (95% CI)	Pre-int	Post-int	Mean % change (95% CI)	T	G	S	TxG	TxS	GxS	TxGxS
V'O2peak (ml.kg^−1^. min^−1^)	M	27.33 (8.02)	36.33 (9.07)	34.6 (5.99 - 63.21)	39.5 (2.12)	42.5 (3.54)	7.51 (−21.03 - 36.05)	< 0.001*; 0.788	0.253; 0.081	< 0.001*; 0.61	< 0.001*; 0.653	0.003*; 0.432	0.014*; 0.32	0.915; 0.001
	F	24 (2.24)	29.14 (3.67)	21.4 (12.06 −30.75)	23.25 (3.96)	22.63 (4.37)	−2.92 (−9.07 - 3.22)							
	Total	25 (4.5)	31.3 (6.27)	25.36 (16.95 - 33.77)	26.5 (7.72)	26.6 (9.3)	−0.84 (−6.49 - 4.82)							
V'O_2_ (L/dk)	M	2.36 (1.95–3.29)	2.77 (2.7–3.73)	23.07 (−10.41 - 56.55)	3.815 (3.47–4.16)	3.685 (3.56–3.81)	−2.91 (−72.84 - 67.02)	0.399; 0.06	0.456; 0.05	< 0.001*; 0.6	0.021*; 0.39	0.008*; 0.36	0.036*; 0.25	0.259; 0.08
	F	1.98 (1.8–2.13)	1.95 (1.76–2.07)	−0.68 (−13.17 - 11.8)	2.025 (1.985–2.385)	1.86 (1.665–2.075)	−13.79 (−10.75 - −6.82)							
	Total	2.04 (1.94–2.31)	2.06 (1.91–2.75)	6.44 (−5.81 - 18.7)	2.155 (2.01–2.65)	1.94 (1.82–2.19)	−11.62 (−18.08 - −5.14)							
V'O_2_ %	M	77.67 (15.95)	97 (11.14)	26.66 (−5.86 - 59.18)	118 (2.83)	117 (11.31)	−0.93 (−65.74 - 63.88)	0.319; 0.062	0.057; 0.208	0.284; 0.071	0.003*; 0.423	0.021*; 0.29	0.210; 0.291	0.588; 0.019
	F	95.14 (13.61)	98.57 (15.04)	4.21 (−8.16 - 16.57)	99.13 (13.43)	87.88 (12.53)	−11.28 (−17.21 - −5.35)							
	Total	89.9 (15.85)	98.1 (13.38)	10.94 (−0.92 - 22.8)	102.9 (14.3)	93.7 (16.94)	−9.21 (−14.93 - −3.49)							
	Total	25.6 (5.02)	31.3 (6.27)	23.2 (12.28 - 34.12)	26.5 (7.72)	26.6 (9.3)	−0.83 (−6.49 - 4.82)							
RER	M	1.13 (0.08)	1.28 (0.06)	14.01 (8.39 - 19.63)	1.11 (0.06)	1.2 (0.07)	8.41 (−98.46 - 115.29)	< 0.001*; 0.668	0.039*; 0.239	0.207; 0.098	0.001*; 0.478	0.002*; 0.457	0.571; 0.02	0.211; 0.096
	F	1.14 (0.07)	1.23 (0.07)	8.37 (4.12 - 12.63)	1.12 (0.05)	1.08 (0.06)	−3.68 (−6.31 - −1.04)							
	Total	1.13 (0.07)	1.25 (0.07)	10.06 (6.66 - 13.47)	1.12 (0.05)	1.1 (0.07)	−1.26 (−6.29 - 3.77)							
V'E (L.min^−1^)	M	82.6 (28.41)	118.53 (30.58)	48.68 (−32.11 - 129.46)	111.15 (11.38)	122 (12.73)	9.75 (7.86 - 11.64)	0.020*; 0.293	0.807; 0.004	< 0.001*; 0.57	0.006*; 0.382	< 0.001*; 0.537	0.112; 0.15	0.594; 0.018
	F	74.84 (10.64)	77.81 (19.42)	4.08 (−15.02 - 23.18)	71.81 (13.99)	57.09 (13.34)	−20.3 (−30.45 - −10.16)							
	Total	77.17 (16.4)	90.03 (29.09)	17.46 (−4.97 - 39.89)	79.68 (21.02)	70.07 (30.09)	−14.29 (−26.16 - −2.43)							
VT peak (L)	M	2.31 (0.22)	2.68 (0.31)	16.43 (−13.88 - 46.74)	2.53 (0.49)	2.59 (0.37)	2.69 (−44.73 - 50.11)	0.083; 0.176	0.938; 0.001	< 0.001*; 0.653	0.025*; 0.276	0.029*; 0.265	0.752; 0.006	0.499; 0.029
	F	1.68 (0.3)	1.74 (0.37)	3.41 (−5.83 - 12.65)	1.73 (0.22)	1.62 (0.29)	−7.07 (−16.11 - 1.96)							
	Total	1.87 (0.41)	2.02 (0.57)	7.31 (−1.12 - 15.76)	1.89 (0.42)	1.81 (0.5)	−5.12 (−12.65 - 2.41)							
BF (breath/min)	M	35.33 (10.41)	43.67 (6.66)	27.61 (−33.85 - 89.07)	45.5 (13.44)	48 (11.31)	6.46 (−52.57 - 65.5)	0.640; 0.014	0.902; 0.001	0.845; 0.002	0.178; 0.11	0.035*; 0.25	0.138; 0.132	0.929; 0.001
	F	45.86 (10.45)	44.86 (5.49)	1.34 (−16.56 - 19.24)	42.25 (9.22)	36.13 (8.82)	−14.39 (−23.75 - −5.03)							
	Total	42.7 (11.08)	44.5 (5.5)	9.22 (−7.51 - 25.94)	42.9 (9.39)	38.5 (9.99)	−10.22 (−19.81 - −0.63)							
BR%	M	46 (34–65)	33 (9–36)	−48.8 (−105.75 - 8.14)	27 (26–28)	19.5 (19–20)	−27.75 (−38.22 - −17.28)	0.735; 0.001	0.384; 0.07	0.209; 0.09	0.020*; 0.31	0.002*; 0.44	0.036*; 0.25	0.954; 0.001
	F	33 (23–43)	37 (19–42)	4.6 (−45.8 - 64)	41.5 (27.5–44)	47 (44–60.5)	47.04 (3 - 91.08)							
	Total	33.5 (32–46)	34.5 (19–40)	−11.42 (−53.94 - 31.09)	37 (26–43)	45 (37–60)	32.08 (−8.09 - 72.25)							
V'E/V'O_2_	M	29.63 (3.33)	35.8 (2.69)	21.83 (−17.85 - 61.51)	27.25 (1.2)	30.9 (1.41)	13.62 (−78.04 - 105.28)	0.027*; 0.27	0.013*; 0.325	0.491; 0.03	0.070; 0.19	0.007*; 0.377	0.605; 0.017	0.612; 0.016
	F	33.93 (3.16)	35.5 (4.52)	4.98 (−6.82 - 16.78)	30.75 (3.31)	27.98 (3.72)	−9.03 (−15.14 - −2.92)							
	Total	32.64 (3.66)	35.59 (3.9)	10.04 (−0.85 - 20.92)	30.05 (3.29)	28.56 (3.54)	−4.5 (−13.09 - 4.09)							
V'E/V'CO_2_	M	26.23 (2.12)	27.83 (1.33)	6.8 (−25.72 - 39.32)	24.5 (0.28)	25.75 (0.21)	5.11 (−13.57 - 23.8)	0.942; 0.001	0.110; 0.152	0.148; 0.126	0.790; 0.005	0.054; 0.212	0.817; 0.003	0.994; 0.001
	F	29.9 (2.04)	28.76 (2.18)	−3.36 (−13.12 - 6.39)	27.56 (3.65)	26.05 (3.6)	−5.42 (−10.44 - −0.41)							
	Total	28.8 (2.63)	28.48 (1.94)	−0.31 (−8.67 - 8.04)	26.95 (3.47)	25.99 (3.18)	−3.32 (−8.28 - 1.65)							
VD/VT ᵃ	M	0.1 (0.07–0.11)	0.04 (0.04–0.1)	−26.93 (−177.12 - 123.27)	0.045 (0.04–0.05)	0.03 (0.03–0.03)	−32.5 (−127.8 - 62.8)	0.163; 0.11	0.154; 0.17	0.162; 0.12	0.339; 0.05	0.055; 0.19	0.020*; 0.3	0.450; 0.04
	F	0.08 (0.06–0.08)	0.07 (0.06–0.09)	−3.79 (−18.14 - 10.57)	0.07 (0.055–0.08)	0.065 (0.055–0.08)	13.13 (−29.07—55.32)							
	Total	0.08 (0.07–0.1)	0.07 (0.05–0.09)	−10.73 (−34.43 - 12.97)	0.065 (0.05–0.08)	0.06 (0.05–0.07)	4 (−30.78—38.78)							

Abbreviations: *HEG* hybrid exercise group, *CG* control group, *Pre-int* pre-intervention, *Post-int* post-intervention, *M* male, *F* female, *T* time, *G* group, *S* sex, *CI* confidence interval. *V̇O₂peak* peak oxygen uptake, *V̇O₂* oxygen uptake, *V̇O₂ %* percent predicted peak oxygen uptake, *RER* respiratory exchange ratio, *V̇E* minute ventilation, *VT peak* peak tidal volume, *BF (breath/min)* breathing frequency, *BR%* breathing reserve (percent), *V̇E/V̇O₂* ventilatory equivalent for oxygen, *V̇E/V̇CO₂* ventilatory equivalent for carbon dioxide, *VD/VT* dead space to tidal volume ratio, *ml.kg⁻*^*1*^*.min⁻*^*1*^ milliliters per kilogram per minute, *L/dk* liters per minute; Descriptives shown with Mean(SD) for three-way repeated measure ANOVA or ᵃMedian(IQR) for Aligned Rank Transform ANOVA. Statistically significant difference at *p < 0.05 level. Peak values represent the highest values obtained during the cardiopulmonary exercise test

### Physical Activity

Table 7 presents changes in physical activity levels assessed by the International Physical Activity Questionnaire (IPAQ) in the hybrid exercise group (HEG) and control group (CG) at baseline and after 16 weeks of intervention, stratified by sex. For total physical activity (MET-min/week), a significant main effect of time (*p* < 0.001; partial η^2^ = 0.951), group (*p* = 0.001; partial η^2^ = 0.495), and sex (*p* < 0.001; partial η^2^ = 0.944) was observed, along with a significant time × group interaction (*p* = 0.003; partial η^2^ = 0.428). In the HEG, total MET values increased markedly from baseline to post-intervention in both males and females, whereas changes in the CG were modest. The mean percentage increase in total MET was substantially greater in the HEG compared with CG across sexes. For walking-related physical activity (MET-min/week), a significant time effect (*p* < 0.001; partial η^2^ = 0.56), sex effect (*p* = 0.001; partial η^2^ = 0.495), and time × group interaction (*p* = 0.003; partial η^2^ = 0.428) were identified, indicating a greater increase in walking activity in the HEG relative to the CG. Categorical IPAQ analysis demonstrated that, at post-intervention, 100% of HEG participants were classified as having a “high” physical activity level, regardless of sex. In contrast, participants in the CG predominantly remained in the “low” or “moderate” physical activity categories, with significant between-group differences observed for males, females, and the total sample (*p* < 0.001).

**Table 7:** IPAQ data of study groups at baseline and after 16 weeks of intervention period

Measure	Sex	HEG			CG			Effect (*p*; partial eta squared)						
		Pre-int	Post-int	Mean % change (95% CI)	Pre-int	Post-int	Mean % change (95% CI)	T	G	S	TxG	TxS	GxS	TxGxS
Walking (MET-min/week)	M	170.5 (74.4)	484 (76.21)	237.78 (−227.5–703.06)	288.75 (58.34)	660 (0)	133.33 (−290.21 - 556.87)	< 0.001*; 0.56	0.112; 0.15	0.001*; 0.495	0.758; 0.006	0.003*; 0.428	0.038*; 0.243	0.725; 0.008
	F	240.43 (118.93)	287.57 (98.47)	78.65 (−98.24–255.54)	220.69 (93.31)	264 (144.38)	50 (−57.53 - 157.53)							
	Total	219.45 (108.63)	346.5 (129.45)	126.39 (−13.23 266.01)	234.3 (89.3)	343.2 (209.98)	66.67 (−19.02 - 152.36)							
Total (MET-min/week)	M	170.67 (74.66)	1924 (76.21)	1206.98 (−361.95–2775.9)	289 (57.98)	660 (0)	133.07 (−287.06 - 553.19)	< 0.001*; 0.951	0.001*; 0.495	< 0.001*; 0.944	0.003*; 0.428	0.038*; 0.243	0.725; 0.008	< 0.001*; 0.951
	F	240.57 (118.87)	1727.57 (98.47)	912.58 (153.15–1672)	220.88 (93.37)	264 (144.38)	49.93 (−57.63 - 157.49)							
	Total	219.6 (108.62)	1786.5 (129.45)	1000.9 (466.35–1535.44)	234.5 (89.33)	343.2 (209.98)	66.56 (−19.12 - 152.24)							
Physical activityᵃ	M	HEG			CG		Post-int-intervention HEG vs CG							
		Pre-int	Post-int		Pre-int	Post-int								
Low		3 (100%)			2 (100%)		0.100							
Moderate						2 (100%)								
High			3 (100%)											
Physical activityᵃ	F	HEG			CG		Post-int-intervention HEG vs CG							
		Pre-int	Post-int		Pre-int	Post-int								
Low		7 (100%)			8 (100%)	8 (100%)	< 0.001*							
Moderate														
High			7 (100%)											
Physical activityᵃ	Total	HEG			CG		Post-int-intervention HEG vs CG							
		Pre-int	Post-int		Pre-int	Post-int								
Low		10 (100%)			10 (100%)	8 (80%)	< 0.001*							
Moderate						2 (20%)								
High			10 (100%)											

Abbreviations: *HEG* hybrid exercise group, *CG* control group, *Pre-int* pre-intervention, *Post-int* post-intervention, *M* male, *F* female, *T* time, *G* group, *S* sex, *CI* confidence interval, *MET* metabolic equivalent of task; Descriptives shown with Mean(SD) for three-way repeated measure ANOVA or ᵃfrequency(%) for Fisher’s Exact test. Statistically significant difference at *p < 0.05 level. Pre-int MET = 0 min/week for all participants (*n* = 20), while Post-int MET = 1440 min/week for all HEG participants (*n* = 10)

## Discussion

The present study evaluated the effects of a remotely supervised, home-based hybrid exercise program initiated three months after bariatric surgery and delivered three days per week for four months. The primary findings indicate that, although bariatric surgery induced marked reductions in body weight, adiposity, lean mass, and RMR in both groups, the hybrid exercise intervention provided additional and clinically meaningful benefits in muscle strength, cardiorespiratory fitness, and physical activity levels.

### Body Composition and Resting Metabolic Rate

Consistent with the rapid postoperative weight-loss phase, significant improvements in body weight, BMI, waist and hip circumferences, and total and regional fat mass were observed in both groups, confirming the dominant effect of surgery. However, these reductions were consistently greater in the Hybrid Exercise Group (HEG), suggesting an additive effect of structured exercise on adiposity-related outcomes, in line with previous reports [4]. Despite these favorable changes, lean body mass and total body water declined modestly in both groups, and RMR decreased similarly in the HEG (− 15.66%) and Control Group (CG; − 14.72%), with no significant between-group differences. These findings are consistent with earlier studies indicating that postoperative reductions in RMR are primarily driven by surgery-related loss of metabolically active tissue rather than modifiable through short-term exercise interventions [22, 46, 47]. Previous meta-analyses have likewise reported limited and inconsistent effects of postoperative exercise on resting energy expenditure [4]. Factors such as delayed initiation of training, insufficient resistance-training volume, and the catabolic milieu associated with rapid weight loss may explain the inability of the present intervention to preserve lean mass or attenuate RMR decline. Collectively, these findings suggest that mitigating postoperative losses in lean mass and RMR may require earlier exercise initiation, higher resistance-training loads, and optimized protein intake.

#### Muscle Strength, Handgrip Strength, and Skeletal Muscle Index

Despite the observed reductions in lean mass and SMI, the hybrid exercise intervention elicited substantial improvements in muscle strength across all upper- and lower-extremity measures. Significant time × group interactions with large effect sizes favored the HEG for all strength outcomes. Improvements were evident in both sexes, although males consistently demonstrated higher absolute strength values. Handgrip strength increased significantly only in the HEG, supporting the functional relevance of the intervention.

Notably, SMI decreased similarly in both groups, indicating that strength gains occurred largely independent of increases in muscle mass. This dissociation suggests that neuromuscular adaptations—such as improved motor unit recruitment, firing rate, and intermuscular coordination—likely played a dominant role in strength development during the intervention period [48]. These findings are particularly relevant given that marked declines in muscle strength were observed during the early postoperative period, especially in the lower extremities, consistent with previous observations in bariatric populations [49]. The pronounced post-intervention gains in lower-limb strength may reflect the involvement of large muscle groups, progressive intensity targets (~ 75% peak HR/VO_₂_peak; Borg 11–15), and high adherence facilitated by supervised online delivery. Overall, these results align with prior studies demonstrating that structured resistance or combined exercise programs can reverse or exceed postoperative strength losses, even in the presence of ongoing lean mass reduction [3, 8, 10, 50, 51].

#### Cardiopulmonary Capacity

Cardiorespiratory fitness improved significantly following the hybrid exercise intervention. Relative VO₂peak demonstrated a strong main effect of time and a significant time × group interaction, indicating superior improvements in the HEG compared with the CG. These changes were observed in both sexes, whereas no meaningful improvements occurred in the CG. Absolute VO_₂_ values were primarily influenced by sex, with males exhibiting higher values at both time points, while percent predicted VO₂peak increased in the HEG and declined in the CG, particularly among females. Improvements in ventilatory parameters, including minute ventilation and peak tidal volume, further suggest enhanced cardiopulmonary efficiency in response to training. Importantly, the exercise program was initiated during a period of ongoing physiological recovery following bariatric surgery. Previous evidence indicates that VO_₂_peak may improve spontaneously during the early postoperative months [49]. Therefore, the greater gains observed in the HEG likely represent an additive effect of structured exercise rather than natural recovery alone. These findings align with prior research demonstrating that combined aerobic and resistance exercise improves VO₂peak and cardiometabolic health in post-bariatric populations [52, 53], as well as with reports demonstrating beneficial effects of higher-intensity aerobic protocols on cardiorespiratory fitness and cardiometabolic risk markers [54].

#### Physical Activity

Physical activity levels increased markedly in the HEG, as evidenced by significant time, group, and sex effects, and a robust time × group interaction for total MET-min/week. Walking-related physical activity also improved significantly, suggesting that participation in structured exercise translated into greater engagement in daily physical activity. At post-intervention, all HEG participants were classified as having a high physical activity level, whereas the CG largely remained in low or moderate categories. These findings underscore the effectiveness of remotely supervised, home-based hybrid exercise models in promoting sustained behavioral change, consistent with previous reports highlighting superior adherence, feasibility, and cost-effectiveness compared with facility-based programs [55–58].

#### Clinical Implications

From a clinical perspective, these findings emphasize the importance of prioritizing functional outcomes alongside weight loss in post-bariatric care. Although preservation of lean mass and RMR remains challenging during the early postoperative period, hybrid exercise programs appear capable of producing substantial improvements in muscle strength, cardiorespiratory fitness, and physical activity levels, all of which are critical determinants of functional capacity and quality of life [1, 59, 60]. To further reduce the risk of sarcopenic obesity, future interventions should consider earlier initiation of exercise, progressive resistance-training volume, adequate protein intake (women ≥ 60 g/day; men ≥ 80 g/day or approximately 1.1 g/kg/day), and careful monitoring of hydration and bone health.

#### Limitations

The sample size met the target determined by the preliminary G*Power analysis, indicating that statistical power was not substantially limited; however, our intention to further increase statistical power by recruiting additional participants could not be achieved due to the emergence of a non-surgical swallowable gastric balloon during the study period, which reduced the number of bariatric surgery procedures. However, the primary aim of our study was to evaluate the effects of the hybrid-type multicomponent exercise model itself. Therefore, in accordance with guideline recommendations, the initiation of exercise—particularly resistance and strength-based training—was scheduled to begin no earlier than six weeks after surgery, ensuring that the intervention adhered to established postoperative safety protocols. Although a standardized nutritional protocol was applied to both the HEG and CG during hospitalization and follow-up, direct monitoring of dietary intake—particularly total caloric consumption and protein supplementation—was not performed, which represents a methodological limitation; moreover, the inability to track patients’ postoperative daily protein intake constitutes an additional limitation, as insufficient protein consumption may have influenced lean mass preservation and muscle strength outcomes.

## Conclusion

Initiated three months after bariatric surgery and performed three days per week over four months, the online, home-based hybrid exercise program yielded several important findings: (1) Produced clinically meaningful improvements in muscular strength across all major body regions, with the greatest gains observed in the lower extremities. (2) Did not increase RMR or prevent early postoperative losses in lean mass, consistent with surgery-related metabolic adaptations occurring during the rapid weight-loss phase. (3) Provided additional benefits to surgery in adiposity-related outcomes (body weight, BMI, regional fat percentages, etc.), although lean mass remained unchanged.(4) The increase in VO_₂_max supports the contribution of exercise to the improvement of cardiometabolic risk factors after surgery. Overall, these findings support the integration of structured and progressively designed hybrid exercise programs into standard postoperative care to enhance functional capacity and quality of life. To better preserve lean mass and mitigate declines in RMR, earlier initiation of exercise, greater resistance-training volume and progression, and careful monitoring of adequate protein intake should be considered.

## Data Availability

No datasets were generated or analysed during the current study.
